# Congress of the Italian Odontological Society

**Published:** 1890-04

**Authors:** 


### CONGRESS OF THE ITALIAN ODONTOLOGICAL SOCIETY.[1]

After a lapse of five years the Sixth Congress of the Italian Odontological Society, that was to have been held in Rome in 1884, was held in Genoa, in the rooms of the "Societa di Conversazioni Scientifiche," on the 1st and 2d of November of last year.

Two meetings were arranged for each day,—from 10 A.M. until 12 M. and from 2 to 4 P.M. The president, Dr. Campani, being absent, the vice-president, Mr. C. W. Dunn, senior, of Florence, took the chair. With the usual formalities the Congress was declared open, and the vice-president pronounced the inaugural address, expressing at the same time a vote of regret for the president's absence on account of ill health. Twelve new members

---

[1] Reported by William Dunn, Jr., D.D.S., Florence, Italy.

were proposed and accepted, after which the Hon. Secretary, Mr. C. S. Bright, of Genoa, proposed some modifications of the then existing statute,—that the Congresses should be held yearly, and that the officers should be appointed from among those present; the modifications were passed after some discussion.

The first thesis was read by Signor Ballerio, of Milan, "On Secondary Dentine and Nodular Calcification." Signor Ballerio finds that in each case the calcific deposit is due to a slow and constant irritation, but whereas it is physiological in the formation of secondary dentine, the process is pathological when it results in nodular calcification.

Dr. Schaffner, of Florence, concurred in Signor Ballerio's ideas. He thinks that if it were possible, by extremely delicate manipulation, to remove the odontoliths from the pulp, the pulp would return to its normal condition. He agrees that such an operation is, for the present, purely theoretical, never having been accomplished. The safer way is slowly to devitalize and extirpate the pulp.

The next paper, by Mr. C. S. Bright, "Whether it be Well to leave Decalcified Dentine or not," was ably handled by the gentleman. He recognizes the advisability of preserving the pulp, even if only a few filaments are left, and therefore would leave decalcified dentine in a cavity if it were protecting a pulp. The decalcified dentine can be well disinfected and often is recalcified later on by the pulp.

Signor Damiano Mela, of Genoa, would make a difference between cases. In some cases decalcified dentine, well disinfected, is the best protection for the pulp, which recalcifies it in time; but in some cases the pulp dies under the decalcified dentine, and an abscess may be established. He does not think disinfection necessary if the cavity be perfectly dry.

Dr. Schaffner thinks that, once a tooth is well formed and calcified, its pulp is not of such great importance to it. He recounts a case of one of his own teeth which, under a nitrophosphate filling, was quiet for seventeen years; later on he suffered from an irritation of the ciliary nerve, which passed away when the tooth was unstopped; refilling the tooth brought on the trouble again, and finally the pulp was devitalized. He is now circumspect in preserving pulps, and prefers to destroy in dubious cases rather than run the risk of a chronic irritation or neuralgia. He mentioned how, according to Miller's latest researches, micrococci precede decay, almost into the healthy dentine, and that therefore it would be extremely difficult to sterilize perfectly any softened dentine.

The meeting was adjourned till the afternoon.

Signor Ballerio, of Milan, presented the next thesis,—"Orthodontia." The greatest number of irregularities treated are in the upper incisors and cuspids, the difficulty was to find a fulcrum from which the force could be exerted, and to find a fixed point on the tooth to be moved on which to exert the force. Complications arose in the shape of the arch and teeth, the articulation, the crowding of teeth, and the state of the gums and mouth; this opened the discussion, which was animated; special cases were described by Dr. Carreras, of Leghorn, and Signor Solari, of Bologna.

The rest of the afternoon was occupied by discussions on the new dental law to be proposed,—that all those desiring to practise dental surgery in Italy must be doctors of medicine. It was decided to telegraph to His Excellency, the Minister of Public Instruction, to thank him for the interest he had taken in raising the standard of the profession in Italy, but to beg that he would consider the modifications the society would offer,—that is to say, to restrict the time of education from seven to three years.

At half-past six the forty and more members met for the social dinner at the Restaurant della Concordia, where good humor reigned throughout. The king, queen, prince, ministers, and president were toasted.

The next morning was occupied by theses,—"Disinfection," by Signor Ballerio, and "Pyorrhœa Alveolaris," by Mr. Bright. Both were discussed; much was said about Signor Damiano Mela's treatment of pyorrhæa alveolaris with finely pulverized sulphate of copper, which seems to be very successful.

A very interesting communication from Professor Miller, of Berlin, was read, on "The Antiseptic Properties of Filling-Materials."

Dr. Miller fully explained two methods of testing the antiseptic property of filling-materials; one by dropping pieces of the filling in a growth of fungi, and the other by using decayed dentine that had been under fillings for some days. As the result of these experiments he comes to the conclusion that copper amalgam has the most marked antiseptic effect of all fillings; and that amalgams are more or less antiseptic in their action according to the proportion of copper that they contain. Some preparations of non-cohesive gold are markedly antiseptic in their action; this action disappeared as soon as the gold was annealed, and for this phenomenon Dr. Miller can as yet find no explanation.

The afternoon was passed in demonstrations. Mr. Dunn showed his adaptation of the ether spray and compressed air for painless operations on and about the teeth.

Dr. Schaffner showed a new saliva-ejector, of his own invention, and an automatic mallet to be worked by compressed air, which acted beautifully.

Signor Mela showed casts of irregularities, most noticeable among which was the model of upper maxillæ with five permanent molars on each side.

Dr. Herbst, of Bremen, sent some very interesting specimens of his fillings by the rotary method, and other specimens of his system of glass crowns and inlays.

Enamel and porcelain inlays were shown by Mr. Dunn, of Florence, and several samples of mechanical work were contributed by Signor Solari and Signor Casotti, of Leghorn.

Drs. Miller and Herbst were unanimously elected honorary members.

The next Congress will be held at Turin.

